# A novel matrix multiplication framework for modeling genotype-by-environment interaction in genomic prediction

**DOI:** 10.3389/fgene.2026.1961511

**Published:** 2026-09-29

**Authors:** Osval A. Montesinos-López, Abelardo Montesinos-López, Cricelio Montesinos-Lopez, José Crossa, Jaime Cuevas, Susanne Dreisigacker, Carlos M. Hernández Suárez, Rodomiro Ortiz

**Affiliations:** 1 Facultad de Telemática, Universidad de Colima, Colima, Mexico; 2 Centro Universitario de Ciencias Exactas e Ingenierías (CUCEI), Universidad de Guadalajara, Guadalajara, Mexico; 3 Consultant, Mexico, Mexico; 4 Centro de Estadística y Ciencias de Datos, Colegio de Postgraduados (COLPOS), Montecillo, Mexico; 5 División de Ciencias, Ingeniería y Tecnologías (DCIT), Universidad Autónoma del Estado de Quintana Roo, Chetumal, Mexico; 6 International Maize and Wheat Improvement Center (CIMMYT), Texcoco, Mexico; 7 Coordinación General de Investigación Científica, Universidad de Colima, Colima, Mexico; 8 Department of Plant Breeding, Swedish University of Agricultural Sciences (SLU), Lomma, Sweden

**Keywords:** genomic selection, genotype by environment interaction, Hadamard product, plant breeding, prediction accuracy

## Abstract

Accurate modeling of genotype-by-environment (G × E) interaction is a central challenge in plant breeding, as it determines how genetic potential is expressed across heterogeneous and changing environments. In genomic prediction, a widely adopted strategy represents G × E interaction through the Hadamard (element-wise) product of genotype and environment covariance matrices, capturing interaction effects via joint similarity. While effective, this formulation may not fully represent the structured complexity of G × E relationships observed in multi-environment trials. In this study, we extend a recently proposed matrix-product framework for covariance construction to explicitly model G × E interaction. Unlike the Hadamard approach, which captures similarity-by-similarity effects, the proposed method derives symmetric kernels from the matrix multiplication of genotype and environment kernels. These kernels retain complementary subsets of matrix-product information, but the numerical upper/lower assignment is tied to record ordering and should not be interpreted as an intrinsic biological direction. Because the direct matrix product and its symmetrized subcomponents are not guaranteed to be valid covariance matrices, positive semidefiniteness must be examined explicitly before the kernels are used as random-effect covariance structures. The proposed framework was evaluated using eleven multi-environment datasets in wheat and rice, comprising diverse traits and experimental structures. On average across datasets, models containing matrix-product-derived kernels showed higher predictive performance than the conventional Hadamard-only formulation, with an average COR difference of up to 13.2% for the combined model. These empirical gains should be interpreted as average predictive differences under the validation scheme used here rather than as a claim of universal or statistically significant superiority. These results show that matrix-product-derived terms can carry predictive information complementary to the conventional Hadamard interaction term. At the same time, the present revision makes explicit two structural limitations that must be considered when interpreting these kernels: positional triangular extraction is record-order dependent, and balanced incidence combined with centered genomic relationships can make the matrix product degenerate or nearly zero. These findings define both the practical scope of the current formulation and priorities for further methodological development.

## Introduction

Understanding G × E interaction is a central challenge in quantitative genetics and plant breeding, as it determines how genetic potential is expressed across diverse environmental conditions. Genotypes often exhibit differential responses to environmental variation, leading to changes in ranking and performance across locations and years. This phenomenon complicates selection decisions and reduces the effectiveness of breeding programs if not properly modeled. Accurately capturing G × E interaction is therefore essential for improving the prediction of genetic values, identifying broadly adapted genotypes, and enhancing genetic gain under heterogeneous and changing environments (Cooper et al., 1999; Crossa, 1990; Crossa et al., 2017).

The statistical modeling of G × E interaction has evolved substantially over the last century. Early approaches relied on analysis of variance to partition phenotypic variation into genetic, environmental, and interaction components (Fisher, 1925), but treated G × E primarily as a nuisance term. Subsequent developments introduced regression-based stability models, such as those of Finlay and Wilkinson (1963) and Eberhart and Russell (1966), which provided a first quantitative characterization of genotype responses across environmental gradients. Building on these foundations, Crossa (1990) provided a comprehensive statistical treatment of G × E interaction, emphasizing the importance of combining stability analysis with predictive accuracy. In parallel, among other researchers, Cornelius et al. (1992) and Cornelius and Crossa (1999) further clarified the role of structured covariance models and multiplicative interaction components, laying the groundwork for modern approaches to modeling G × E.

Later developments introduced multivariate and mixed-model methodologies, such as additive main effects and multiplicative interaction (AMMI) models (Gauch, 1988) and genotype main effect plus G × E interaction (GGE) biplots (Yan et al., 2000), which enabled simultaneous modeling of main effects and interaction patterns. These approaches provided both interpretability and predictive capacity and remain widely used in the analysis of multi-environment trials.

With the advent of genomic selection (Meuwissen et al., 2001), the focus shifted toward predictive modeling, where the objective is to estimate the performance of untested genotypes using genome-wide marker data. However, early genomic prediction models often ignored G × E interaction or treated it in simplified ways, resulting in reduced prediction accuracy in multi-environment trials.

To address this limitation, linear mixed models incorporating structured covariance matrices were developed (Crossa et al., 2010; Burgueño et al., 2012). These developments build on mixed-model foundations and include the integration of genomic information, environmental covariates, crop-growth models, machine-learning methods, and high-throughput phenotypes in multi-environment prediction (Henderson, 1984; Heslot et al., 2014; Cooper et al., 2016; Montesinos-López et al., 2016; Montesinos-López et al., 2018; Messina et al., 2018; Washburn et al., 2020; Crossa et al., 2021). These developments were further consolidated by the integration of genomic prediction and G × E modeling frameworks, which combine marker-based relationships with environmental information to improve prediction accuracy in multi-environment trials (Crossa et al., 2017).

A widely adopted strategy for modeling G × E interaction in genomic prediction is based on the Hadamard (element-wise) product of genotype and environment covariance matrices. This formulation captures interaction effects through joint similarity: interaction strength is large when genotypes are genetically similar, and environments are environmentally similar. The approach is attractive due to its biological interpretability, statistical parsimony, and computational efficiency, and has been successfully applied in multi-environment genomic prediction models (Jarquín et al., 2014; Cuevas et al., 2017). Nevertheless, the Hadamard product represents only one way of combining genotype and environment information and implicitly assumes that interaction effects can be fully characterized through joint similarity structures.

Recent developments in kernel methods and multi-source data integration suggest that alternative combinations of covariance structures may capture additional aspects of complex biological interactions. Cuevas et al. (2025) proposed a framework based on matrix multiplication for integrating pedigree and genomic information, from which two complementary covariance matrices are derived and modeled as separate random effects. This approach has shown improved predictive performance when integrating multiple sources of biological information, including genomics, transcriptomics, metabolomics, and phenomics (Montesinos-López et al., 2025a; Montesinos-López et al., 2025b). However, despite its success in multi-source integration, this framework has not yet been explored for modeling G × E interaction.

The key contribution of this study is the adaptation of the framework of Cuevas et al. (2025) to explicitly model G × E interaction. While originally developed for integrating multiple sources of biological information, we evaluate whether matrix-product-derived covariance terms can add predictive information to conventional G × E genomic models. Unlike the Hadamard product, which represents joint genotype-environment similarity element by element, the matrix product combines the two relationship structures through ordinary matrix multiplication. The upper- and lower-triangular symmetrizations used here retain complementary numerical subsets of that product; however, because this partition is positional, it should not be interpreted as an intrinsic biological direction.

Building on this idea, we extend the framework of Cuevas et al. (2025) to explicitly model G × E interaction in genomic prediction. We construct two covariance matrices from the upper and lower triangular components of the matrix product between genotype and environment kernels and incorporate them as random effects in linear mixed models. This leads to a family of models that either replace or augment the conventional Hadamard-based interaction term. We hypothesize that combining both approaches provides a more complete representation of G × E structure.

To evaluate this hypothesis, we analyze eleven wheat and rice multi-environment datasets. The empirical objective is to compare the predictive performance of the conventional Hadamard formulation with models that contain one or both matrix-product-derived kernels. We report the observed average differences without assuming that the matrix-product formulation must be superior in every dataset, trait, environment, or metric.

Overall, this work provides a new perspective on modeling G × E interaction in genomic prediction by introducing complementary kernel structures that enhance predictive accuracy. The proposed framework is general, flexible, and readily implementable within standard mixed-model software, offering a practical and effective tool for modern plant breeding programs operating under complex environmental conditions.

## Materials and methods

### Datasets

#### Datasets 1–3. Wheat

The datasets used in this study correspond to elite yield trial (EYT) cycles from the Global Wheat Program of the International Maize and Wheat Improvement Center (CIMMYT). Specifically, they include the 2013–2014, 2014–2015, and 2015–2016 cycles, hereafter referred to as EYT_1, EYT_2, and EYT_3, respectively. These trials were conducted across four cropping seasons, each evaluated in four or five environments.

The study included 776 lines in EYT_1,775 in EYT_2, and 964 in EYT_3. Trials were conducted using an alpha lattice design, with 39 trials in total. Each trial consisted of 28 lines and two checks, arranged into six blocks with three replications. Across the datasets, several traits were evaluated depending on the environment and lines. For consistency, four traits were analyzed for each line in every environment: (i) days to heading, defined as the number of days from germination until 50% spike emergence, (ii) days to maturity, defined as the number of days from germination until 50% physiological maturity, or when 50% of spikes had lost their green color, (iii) plant height, and (iv) grain yield (GY). Further details regarding the experimental design and the computation of best linear unbiased estimates are provided in Juliana et al. (2018).

Lines from EYT_1 were evaluated in four environments: bed planting with five irrigations (Bed5IR), early heat (EHT), flat planting with five irrigations (Flat5IR), and late heat (LHT). EYT_2 was assessed in five environments: bed planting with two irrigations (Bed2IR), Bed5IR, EHT, Flat5IR, and LHT. Similarly, EYT_3 was evaluated in five environments: Bed2IR, Bed5IR, Flat5IR, flat planting with drip irrigation (FlatDrip), and LHT.

Genome-wide markers for the 2,515 lines (776 + 775 + 964) were obtained through genotyping-by-sequencing [GBS; Elshire et al. (2011); Poland et al. (2012)] at Kansas State University using the Illumina HiSeq2500 platform. From an initial set of 34,900 markers, 2,038 were retained after quality filtering. Missing marker data were imputed with LinkImpute (Money et al., 2015), implemented in TASSEL v5 (Bradbury et al., 2007). Lines with more than 50% missing data were excluded, resulting in a final dataset of 2,515 lines across the three EYT cycles.

#### Dataset 4. Rice indica

The Indica dataset (Monteverde et al., 2019) is a rice resource that includes phenotypic records for four traits: Percentage of Head Rice Recovery (PHR, g), measured as the weight of intact milled kernels from a 100 g rough rice sample; Grain Yield (GY, kg/ha) of paddy rice; Plant Height (PH, cm), recorded from the soil surface to the tip of the flag leaf; and Grain Chalkiness (GC, %), quantified as the proportion of chalky kernels in a 50 g subsample of total milled rice. These traits were collected across three growth stages—vegetative, reproductive, and maturation—over three environments (2010, 2011, and 2012). In each environment, 327 genotypes were evaluated, resulting in a total of 981 observations, with each genotype tested once per environment. After quality control, 16,383 SNP markers were retained and coded as 0, 1, or 2. Further details are provided in Table 1 and in Monteverde et al. (2019).

**TABLE 1:** Description of the datasets.

Dataset number	Dataset (crop and number)	Cultivars (number)	Markers (number)	Traits (number)
Dataset 1	EYT_1	776	2,038	4
Dataset 2	EYT_2	775	2,038	4
Dataset 3	EYT_3	964	2,038	4
Dataset 4	Indica	327	16,383	4
Dataset 5	Japonica	320	16,383	4
Dataset 6	Wheat_1	1301	5741	1
Dataset 7	Wheat_2	1403	5741	1
Dataset 8	Wheat_3	1275	5741	1
Dataset 9	Wheat_4	1388	5741	1
Dataset 10	Wheat_5	1398	5741	1
Dataset 11	Wheat_6	1277	5741	1

#### Datasets 5. Rice japonica

The Japonica dataset consists of 320 genotypes from the tropical rice Japonica population. It was evaluated for the same four traits as the Indica dataset—Plant Height (PH), Percentage of Head Rice Recovery (PHR), Grain Yield (GY), and Grain Chalkiness (GC)—across five environments from 2009 to 2013. In total, 1,051 observations were recorded, although the dataset is unbalanced. Each genotype was also genotyped with 16,383 SNP markers retained after quality control, coded as 0, 1, or 2. Additional details are available in Monteverde et al. (2019).

#### Datasets 6–11 wheat

Six data sets of spring wheat lines tested for grain yield from CIMMYT’s first-year yield trials (YT), as described in Ibba et al. (2020), were used in this study:

- Wheat_1 (YT2013-14/EYT2014-15): 1,301 lines from the 2013–14 YT and 472 lines from the 2014–15 EYT.
- Wheat_2 (YT2014-15/EYT2015-16): 1,337 lines from the 2014–15 YT and 596 lines from the 2015–16 EYT.
- Wheat_3 (YT2015-16/EYT2016-17): 1,161 lines from the 2015–16 YT and 556 lines from the 2016–17 EYT.
- Wheat_4 (YT2016-17/EYT2017-18): 1,372 lines from the 2016–17 YT and 567 lines from the 2017–18 EYT.
- Wheat_5 (YT2017-18/EYT2018-19): 1,386 lines from the 2017–18 YT and 509 lines from the 2018–19 EYT.
- Wheat_6 (YT2018-19/EYT2019-20): 1,276 lines from the 2018–19 YT and 124 lines from the 2019–20 EYT.

All lines were genotyped using genotyping-by-sequencing (GBS). Marker polymorphisms were called with the TASSEL v5 GBS pipeline, applying a minor allele frequency (MAF) threshold of 0.01 for SNP discovery. A total of 6,075,743 unique tags were aligned to the wheat reference genome (RefSeq v.1.0; IWGSC 2018), achieving an alignment rate of 63.98%. After filtering SNPs for >80% homozygosity, *p* < 0.001 based on Fisher’s exact test, and χ^2^ values below the critical threshold of 9.2, 78,606 GBS markers passed at least one of these criteria. Additional filtering retained markers with <50% missing data, MAF > 0.05, and <5% heterozygosity. We imputed missing data using the expectation–maximization algorithm implemented in the R package rrBLUP (Endelman, 2011).

Table 1 outlines the general characteristics of the 11 public datasets used in this study. Nine datasets correspond to wheat trials, and two correspond to rice sets (Indica and Japonica). The public repository at https://github.com/osval78/New_GE_Framework contains the phenotypic and genomic data for all datasets, along with the code used to implement the proposed methodology.

### Statistical models

#### Model A

The predictive response model was defined as shown in Equation 1:

$$Y_{\text{ij}}=\mu +E_{i}+g_{j}+\varepsilon_{\text{ij}}$$ (1)

where $Y_{\text{ij}}$ denotes the response variable measured for genotype j in environment i ; μ denotes the general mean or intercept; $E_{i}$ , i=1,…,I , denotes the random effects of environments, distributed as $E={\left(E_{1},\ldots ,E_{I}\right)}^{T}∼N_{I}\left(0,\sigma_{E}^{2}I_{I}\right)$ , with $I_{I}$ being the identity matrix of order I ; $g_{j},j=1,\ldots ,J$ , are the random effects of lines; and $\epsilon_{\text{ij}}$ denotes the random error terms, assumed normally distributed with mean 0 and variance $\sigma^{2}$ . Furthermore, it was assumed that $g={\left(g_{1},\ldots ,g_{J}\right)}^{T}∼N_{J}\left(0,\sigma_{g}^{2}G\right)$ , where G denotes the linear kernel computed as $G=\frac{XX^{T}}{p}$ . This matrix is known as the genomic relationship matrix (VanRaden, 2008) in plant and animal breeding, since it is computed from a standardized matrix of SNPs ( $\left.X\right)$ **,** coded as 0, 1, and 2, of dimension J×p . The matrix G is considered known prior to the modelling process.

Let y be the stacked vector of all observations $Y_{\text{ij}}$ (of length n = IJ). A common matrix formulation of Equation 1 is given in Equation 1a:

$$y=1_{n}\mu +Z_{E}E+Z_{g}g+\varepsilon$$ (1a)

where $1_{n}$ is the n×1 vector of ones, $Z_{E}$ is the n×I incidence matrix linking observations to environment, $Z_{g}$ is the n×J incidence matrix linking observations to genotypes (lines), and ε is a vector of residuals distributed as $\varepsilon ∼N_{n}\left(0,\sigma^{2}I_{n}\right)$ .

#### Model B

Model B extends Model A by including G × E interaction effects, as shown in Equation 2:

$$y=1_{n}\mu +Z_{E}E+Z_{g}g+g_{E}+\varepsilon$$ (2)

All terms were defined as in Model A, except the interaction term gE which denotes the G×E interaction effects, $\text{gE}={\left(gE_{11},\ldots ,gE_{1J},\ldots ,gE_{IJ}\right)}^{T}$ , that were modeled as a multivariate normal distribution $\text{gE}∼N_{IJ}\left(0,\sigma_{\text{gE}}^{2}\left(K_{g}{}^{\circ}K_{E}\right)\right)$ , where ${K_{g}=Z}_{g}GZ_{g}^{T}$ , with $Z_{g}$ the n×J incidence matrix for additive genetic effects, the variance component $\sigma_{\text{gE}}^{2}$ corresponds to the G×E interaction, $K_{E}=\frac{Z_{E}Z_{E}^{T}}{I}$ represents the linear kernel of the environments, where $Z_{E}$ is the n×I incidence matrix for the environmental effects. ° denotes the Hadamard (element-wise) product. The Hadamard product $K_{g}{}^{\circ}K_{E}$ captures the variance-covariance structure of the **G×E** interaction effects because: (a) ${\left(K_{g}{}^{\circ}K_{E}\right)}_{ij}={K_{g}}_{ij}{K_{E}}_{ij}$ is large only when both genetic similarity $\left({K_{g}}_{ij}\right)$ and environmental similarity ( ${K_{E}}_{ij}$ ) are large, where ${K_{g}}_{ij}$ denotes $\left(i,j\right)$ the entry of a matrix $K_{g}$ **,** and (b) it captures joint similarity, modeling effects that are specific to particular genetic-environmental combinations. Since both $K_{g}$ and $K_{E}$ are symmetric and positive semi-definite (PSD) variance-covariance matrices (see proof in Supplementary Appendix A) their Hadamard product ( $\left.K_{g}{}^{\circ}K_{E}\right)$ is also PSD, ensuring a valid variance-covariance matrix.

#### Model C

Model C extends Model A by incorporating an alternative structure for the G × E interaction:

$$y=1_{n}\mu +Z_{E}E+Z_{g}g+g_{\text{EC}}+\varepsilon$$ (3)

All components match those in Model A, except for the term gEC that denotes the G×E interaction effects. Let $\text{gEC}={\left(g{\text{EC}}_{11},\ldots ,g{\text{EC}}_{1J},\ldots ,g{\text{EC}}_{IJ}\right)}^{T}$ . These interaction effects are modeled using a multivariate normal distribution, $\text{gEC}∼N_{IJ}\left(0,\sigma_{CC}^{2}K^{CC}\right)$ , where $\sigma_{CC}^{2}$ is the variance component associated with this interaction, and $K^{CC}$ is a matrix derived from the matrix multiplication product of the environmental $K_{E}$ and $K_{g}$ genetic kernels. Let $M=K_{E}K_{g}$ , then decompose M into three mutually exclusive parts:

- Upper-triangular off-diagonal part (U)

$$U=\left\{\;M\text{ij if }i\;<\;j;\;0\text{ otherwise }\right\}$$

- Diagonal part (D)

$$D=\left\{\;M\text{ii if }i=j;\;0\text{ otherwise }\right\}$$

- Lower-triangular off-diagonal part (L)

$$L=\left\{\;M\text{ij if }i\;>\;j;\;0\text{ otherwise }\right\}$$

$$M=L+D+U;\;K_{\text{CC}}=U+D+U^{T}$$

#### Model D

Model D extends model A by incorporating an alternative structure for the G × E interaction:

$$y=1_{n}\mu +Z_{E}E+Z_{g}g+g_{\text{EP}}+\varepsilon$$ (4)

All components are as defined previously, except for the term gEP , which denotes an alternative G×E interaction effect. Let $\text{gEP}={\left(g{\text{EP}}_{11},\ldots ,g{\text{EP}}_{1J},\ldots ,g{\text{EP}}_{IJ}\right)}^{T}$ . These effects are modeled using a multivariate normal distribution $\text{gEP}∼N_{IJ}\left(0,\sigma_{PP}^{2}K^{PP}\right)$ , where $\sigma_{PP}^{2}$ is the variance component associated with the random effect gEP **.**

Using the same decomposition of the matrix multiplication product $M=K_{E}K_{g}$ into **L**, **D**, and **U,** the covariance matrix for Model D is based on the lower-triangular component:

$$K_{\text{PP}}=L+D+L^{T}$$

Model D analogously symmetrizes the lower-triangular part together with the diagonal. The resulting matrix is symmetric, but its positive semidefiniteness must be checked and, when required, enforced by the PSD projection described in Supplementary Material 1. As for Model C, “lower” denotes numerical position under the chosen record ordering rather than a biologically intrinsic direction.

#### Model E

Model E extends the previous models by incorporating both upper and lower triangular interaction structures:

$$y=1_{n}\mu +Z_{E}E+Z_{g}g+g_{\text{EC}}+g_{\text{EP}}+\varepsilon$$ (5)

All terms correspond to those specified in the preceding models. As shown in Equation 5, this model incorporates the interaction terms defined in Equations 3, 4, aiming to capture the complex interaction structure represented by the upper and lower triangular matrices derived from the matrix product of the $K_{E}$ and $K_{g}$ linear kernels.

#### Model F

Model F incorporates all previously defined components of the G × E interaction, as shown in Equation 6:

$$y=1_{n}\mu +Z_{E}E+Z_{g}g+g_{E}+g_{\text{EC}}+g_{\text{EP}}+\varepsilon$$ (6)

All terms remain consistent with those used in the earlier models. Model F combines the conventional Hadamard-based G × E kernel (Model B) with the two matrix-product-derived kernels used in Models C and D. The Hadamard term models joint genotype-environment similarity, whereas the additional terms retain complementary subsets of the ordinary matrix product under the specified record ordering. We therefore interpret them as alternative numerical covariance representations, not as evidence of inherent biological direction or asymmetry.

Permutation and ordering limitation. If the same observations are simultaneously permuted in rows and columns, the full matrix product transforms consistently with that relabeling, but extracting a numerical upper or lower triangle does not in general commute with an arbitrary permutation. Consequently, the separate triangular kernels used in the present formulation can depend on record ordering even when the underlying biological data are unchanged. The released implementation sorts records before kernel construction to make the computational convention reproducible, but sorting is a convention and is not a proof of permutation equivariance. The present paper therefore restricts its claims to the predictive behavior of these matrix-product-derived terms under the stated construction. Developing a decomposition whose individual components are permutation-equivariant is an important, separate methodological direction.

$$M^{*}=\text{QM}Q^{T},U\left(\text{QM}Q^{T}\right)\neq QU\left(M\right)Q^{T}$$

Balanced-incidence limitation. The magnitude and rank of the product also depend on the genotype-by-environment incidence structure and on centering of the genomic kernel. Under a complete balanced design, the cross-incidence structure can reduce to an all-ones form. If the genomic relationship matrix is constructed from centered markers, its row/column sums can be zero (or numerically close to zero), in which case the relevant matrix product may be degenerate or nearly zero. Thus, signal in the product can depend strongly on imbalance, replication, missing genotype-environment cells, and the kernel centering used. A derivation and diagnostic interpretation are provided in Supplementary Material 2.

Low-rank interpretation. A balanced design can therefore produce a matrix product with rank one or otherwise very low effective rank. Low rank does not imply absence of predictive utility: a dominant low-dimensional direction can still improve prediction when it aligns with reproducible phenotype-associated covariance. However, predictive improvement from such a kernel does not by itself establish that the kernel represents a rich or uniquely identifiable G × E interaction. We therefore interpret predictive performance jointly with experimental balance, matrix rank, and eigenvalue concentration rather than using prediction gain alone as evidence of biological interaction complexity.

$$\text{rank}\left(M\right)\leq \min\left\{\text{rank}\left(K_{E}\right),\text{rank}\left(K_{G}\right)\right\}$$

**Z**_**E**_^**T**^ **Z**_**g**_ = **J**, **M** = **K**_**E**_ **K**_**g**_; centered markers may imply **K**_**g**_ **1** = **0**

All models were implemented in the R statistical software (R Core Team, 2025) using the BGLR package (Pérez and De Los Campos, 2014), a Bayesian implementation; we used 10,000 iterations, with a burn-in of 2,500 iterations and a thinning interval of 5.

PSD validation and correction. For each matrix-product-derived symmetric kernel **K**, we compute an eigendecomposition **K** = VΛV^T^. Eigenvalues below a numerical tolerance are treated as negative and replaced by zero, yielding K_+_ = VΛ_+_V^T^ with Λ_+_ = diag[max(λᵢ,0)]. The corrected matrix is then symmetrized numerically and scaled using the same normalization rule used for model fitting. This operation is a projection onto the PSD cone; it can alter the diagonal, so diagonal preservation is not claimed. The exact algorithm, tolerance rule, correction magnitude to be reported, and recommended spectral diagnostics are given in Supplementary Material 1.

$$K=V\Lambda V^{T},\Lambda_{+}=\text{diag}\left\{\max\left(\lambda_{i},0\right)\right\},K_{+}=V\Lambda_{+}V^{T}$$

It is important to note that Models A and B correspond to conventional specifications: Model A includes only main effects, whereas Model B incorporates both main effects and G × E interactions modeled via the Hadamard product. In contrast, Models C through F represent the proposed extensions, obtained by adapting the matrix-product-based framework of Cuevas et al. (2025) to the specific context of G × E interaction. Model C extends Model A by including an additional random effect constructed from the upper triangular component of the matrix product between $K_{E}$ and $K_{g}$ . Similarly, Model D extends Model A by incorporating a random effect based on the lower triangular component of the same matrix product. Model E further augments Model A by including two random effects, derived from both the upper and lower triangular components of the matrix product between $K_{E}$ and $K_{g}$ . Finally, Model F builds upon Model B by adding these two random effects, thereby combining the conventional Hadamard-based interaction term with those derived from matrix multiplication.

From these formulations, we hypothesize that Model F will outperform Model B, as it not only retains the conventional G × E interaction term based on the Hadamard product but also incorporates additional structure through the two random effects derived from matrix multiplication. Moreover, we expect Model F to achieve higher prediction accuracy than Models C, D, and E, since these models rely exclusively on the matrix-product-based components—either partially (Models C and D) or fully (Model E)—while omitting the conventional interaction term. Compliance with the assumptions of the proposed models is provided in the Supplementary Materials.

### Cross validation and evaluation metrics

To evaluate and compare the predictive performance of the models, we employed a cross-validation strategy that simulated the scenario of untested lines in tested environments (ULTE). This approach involved five random partitions of all lines in the dataset, with each partition using 50% of the lines for training and the remaining 50% of lines for testing (Alemu et al., 2024; Burgueño et al., 2012). Predictive performance was summarized by Pearson correlation (COR) and PM_10. PM_10 is defined as 100 × |T10,obs ∩ T10,pred|/ |T10,obs|, where T10,obs and T10,pred are the observed and predicted top-decile sets within the corresponding test unit. Ties at the cutoff must be handled by a fixed reproducible rule in the analysis code. Standard errors should be calculated over the same paired resampling units used to compare models. Because the current manuscript reports averages from the original repeated partitions and does not include a formal paired significance test for every model contrast, terms such as “significantly” and “consistently outperformed” are avoided.

Kernel overlap and variance-component interpretation. Models E and F contain multiple kernels derived from the same underlying genotype and environment information. Their random-effect covariance structures can therefore be correlated or nearly collinear. Individual variance components should not automatically be interpreted as uniquely explained, orthogonal portions of G × E variation, and the sum VGE + VGEC + VGEP is used here only as a descriptive total of fitted variance-component magnitudes. Kernel correlations, numerical ranks, eigenvalue spectra, and condition diagnostics should accompany the reproducibility, together with MCMC convergence diagnostics for the fitted BGLR models.

## Results

We provide the results in five sections; in sections from one to four we provide the results for data sets EYT_1, Indica, Japonica and Wheat_1, while in section five we provide the results across the eleven data sets under study. The remaining results are given in Supplementary Appendix B.

### EYT_1 dataset

Regarding the proportion of variance explained by each component in the models for this dataset, the largest proportion of explained variance across all models corresponds to the environment component (VEnv= $\sigma_{E}^{2}$ ), followed by the genotype component (VG= $\sigma_{g}^{2}$ ) (Table 2). For the G × E components (VGE= $\sigma_{\text{gE}}^{2}$ , VGEC= $\sigma_{CC}^{2}$ , and VGEP= $\sigma_{PP}^{2}$ ), only Model F accounted for a larger proportion (35% increase) of the total variability explained (VGE_A = VGE + VGEC + VGEP) compared with Model B, which included the conventional G × E interaction term.

**TABLE 2:** Proportion of total variance across traits in EYT_1 dataset explained for each variance component in each of the evaluated models. VGE_A = VGE + VGEC + VGEP, which denotes the total proportion of variance explained by the G × E interaction. VEnv= $\sigma_{E}^{2}$ , VG= $\sigma_{g}^{2}$ , VGE= $\sigma_{\text{gE}}^{2}$ , VGEC= $\sigma_{CC}^{2}$ , VGEP= $\sigma_{PP}^{2}$ and VE= $\sigma^{2}.$

Dataset	Methods	VEnv	VG	VGE	VGEC	VGEP	Ve	VGE_A
EYT_1	A	0.719	0.188	-	-	-	0.093	0.000
EYT_1	B	0.695	0.208	0.034	-	-	0.064	0.034
EYT_1	C	0.711	0.190	-	0.011	-	0.088	0.011
EYT_1	D	0.712	0.192	-	-	0.009	0.087	0.009
EYT_1	E	0.706	0.195	-	0.014	0.011	0.074	0.025
EYT_1	F	0.677	0.218	0.028	0.010	0.008	0.059	0.046

In terms of COR, the best prediction performance was achieved by Model F (COR = 0.395), followed by Model E (COR = 0.378), which was 4.5% lower than Model F (Figure 1A). In contrast, the poorest performance was observed in Model A (COR = 0.317; Figure 1A), representing a 24.6% decrease compared to Model F, while Model D ranked second lowest with a COR of 0.342 (15.5% lower than Model F).

**FIGURE 1:**
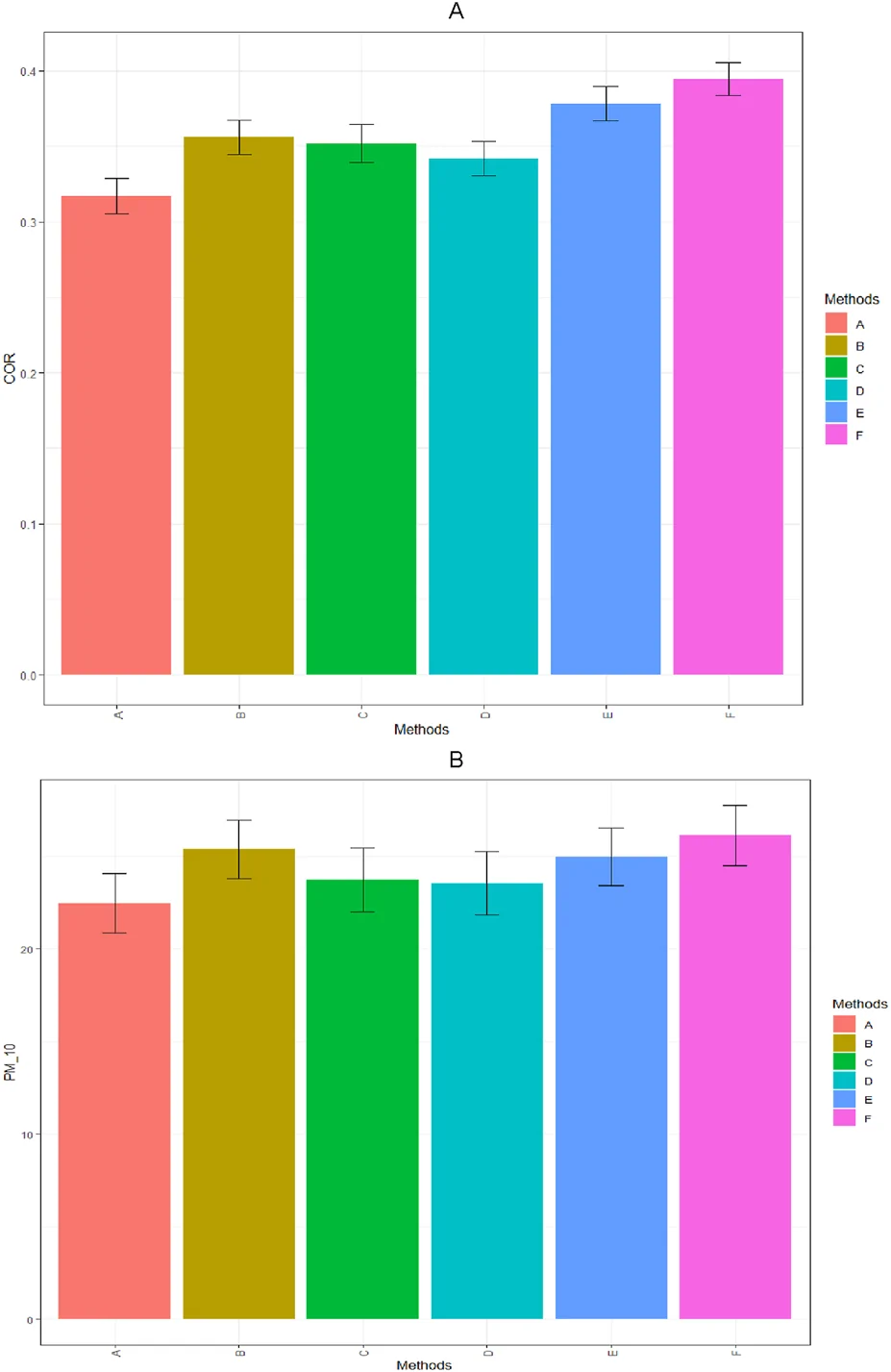
Prediction performance of the six evaluated methods on the EYT_1 dataset, measured by average Pearson’s correlation [COR; **(A)**] and the proportion of test items whose predicted top 10% lines match the true top 10% [PM_10, **(B)**].

Regarding PM_10 Model F exhibited the highest predictive performance (PM_10 = 26.122), followed closely by Model B (PM_10 = 25.369), which was 3.0% lower than that of Model F (Figure 1B). Conversely, Model A showed the lowest performance (PM_10 = 22.452; Figure 1B), reflecting a 16.3% reduction relative to Model F. Model D had the second-lowest value, with a PM_10 of 23.452, corresponding to an 11.0% decrease compared to Model F. More details of the prediction performance for this dataset are given in Supplementary Appendix A, Supplementary Table A2.

### Indica dataset

The genotype component contributed the highest share of explained variance in all models, followed by the error component (Table 3). Among the G × E components (VGE, VGEC, and VGEP), only Model F captured a greater proportion of the total variability—an increase of 17.141%—compared with Model B, which relied on the conventional G × E interaction term.

**TABLE 3:** Proportion of total variance across traits in the Indica dataset explained for each variance component in each of the evaluated models. VGE_A = VGE + VGEC + VGEP, which denotes the total proportion of variance explained by the G × E interaction. VEnv= $\sigma_{E}^{2}$ , VG= $\sigma_{g}^{2}$ , VGE= $\sigma_{\text{gE}}^{2}$ , VGEC= $\sigma_{CC}^{2}$ , VGEP= $\sigma_{PP}^{2}$ and VE= $\sigma^{2}.$

Dataset	Methods	VEnv	VG	VGE	VGEC	VGEP	Ve	VGE_A
Indica	A	0.056	0.493	-	-	-	0.450	0.000
Indica	B	0.048	0.541	0.105	-	-	0.306	0.105
Indica	C	0.056	0.525	-	0.027	-	0.392	0.027
Indica	D	0.047	0.538	-	-	0.023	0.392	0.023
Indica	E	0.051	0.552	-	0.031	0.030	0.337	0.061
Indica	F	0.042	0.566	0.088	0.022	0.013	0.269	0.123

With respect to COR, the highest predictive accuracy was obtained by Model F (COR = 0.405), followed by Model E (COR = 0.399), which was 1.5% lower than that of Model F (Figure 2A). In contrast, Model A recorded the weakest performance (COR = 0.359; Figure 2A), representing a 12.8% decline relative to Model F, while Model C ranked second lowest with a COR of 0.372, reflecting an 8.9% reduction compared to Model F.

**FIGURE 2:**
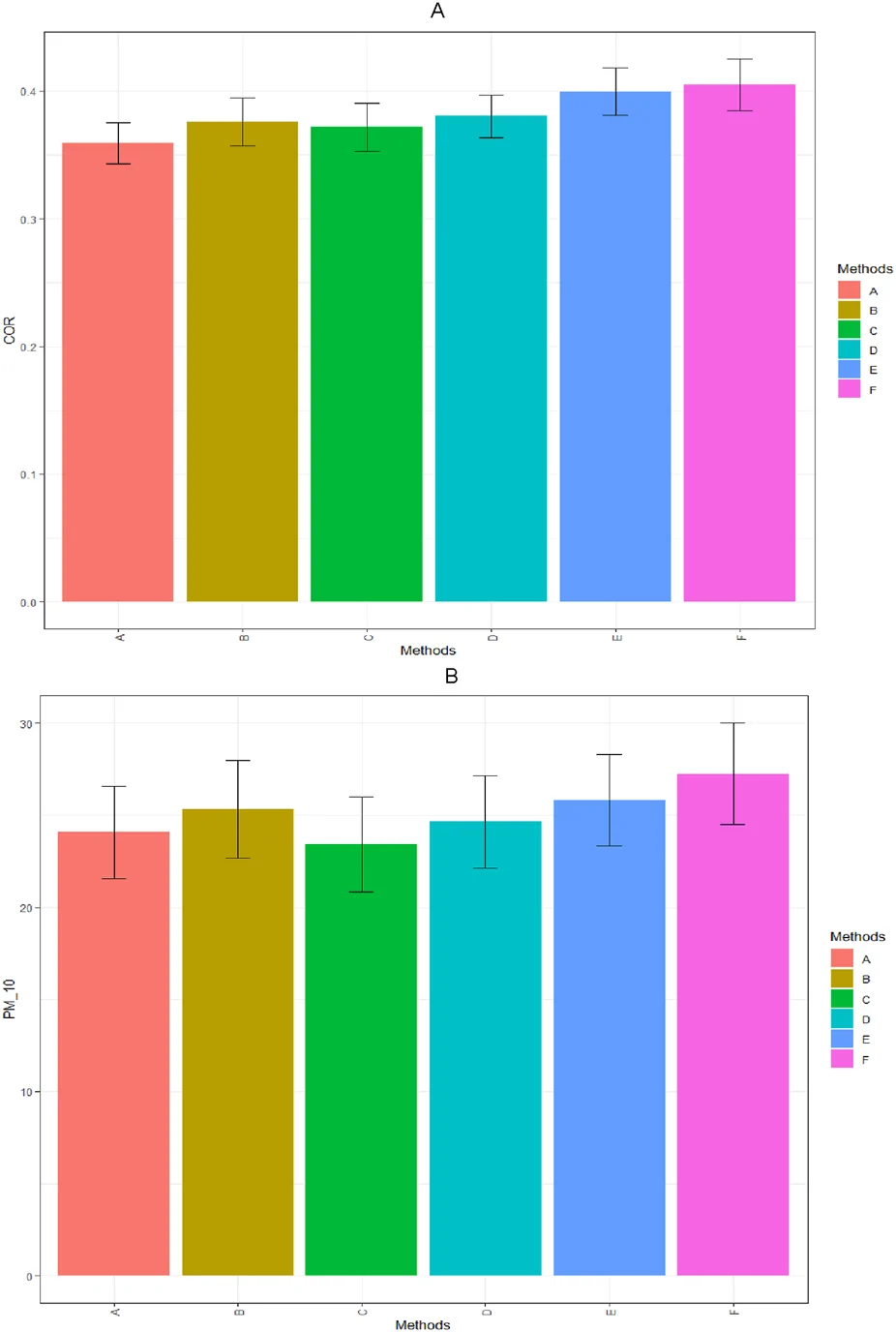
Prediction performance of the six evaluated methods on the Indica dataset, measured by average Pearson’s correlation [COR; **(A)**] and the proportion of test items whose predicted top 10% lines match the true top 10% [PM_10, **(B)**].

In relation to PM_10, Model F delivered the strongest predictive outcome (PM_10 = 27.255), followed closely by Model E (PM_10 = 25.833), which was 5.5% below that of Model F (Figure 2B). On the other hand, Model C registered the lowest result (PM_10 = 23.431; Figure 2B), amounting to a 16.3% decrease in comparison with Model F. Model A followed as the second-lowest performer, with a PM_10 of 24.069, indicating a 13.2% reduction relative to Model F. More details of the prediction performance for this dataset are given in Supplementary Appendix A, Supplementary Table A2.

### Rice Japonica dataset

Across all models, the environment component accounted for the largest proportion of explained variance, followed by the genotype component (Table 4). For the G × E components (VGE, VGEC, and VGEP), only Model F explained a higher share of the total variability—showing a 12.162% increase—relative to Model B, which included the standard G × E interaction term.

**TABLE 4:** Proportion of total variance in the Japonica dataset, explained for each variance component in each of the evaluated models. VGE_A = VGE + VGEC + VGEP, which denotes the total proportion of variance explained by the G × E interaction. VEnv= $\sigma_{E}^{2}$ , VG= $\sigma_{g}^{2}$ , VGE= $\sigma_{\text{gE}}^{2}$ , VGEC= $\sigma_{CC}^{2}$ , VGEP= $\sigma_{PP}^{2}$ and VE= $\sigma^{2}.$

Dataset	Methods	VEnv	VG	VGE	VGEC	VGEP	VE	VGE_A
Japonica	A	0.460	0.312	-	-	-	0.227	0.000
Japonica	B	0.422	0.347	0.074	-	-	0.156	0.074
Japonica	C	0.460	0.309	-	0.016	-	0.215	0.016
Japonica	D	0.463	0.312	-	-	0.016	0.208	0.016
Japonica	E	0.459	0.313	-	0.014	0.014	0.199	0.029
Japonica	F	0.430	0.349	0.061	0.011	0.010	0.138	0.083

Concerning COR, Model F achieved the greatest predictive accuracy (COR = 0.421), followed by Model E (COR = 0.413), which was 1.9% lower than model F (Figure 3A). Conversely, model A exhibited the lowest performance (COR = 0.397; Figure 3A), reflecting a 6.0% decrease compared to Model F. Models C and D tied for the second-lowest performance, each with a COR of 0.404, representing a 4.2% reduction relative to Model F.

**FIGURE 3:**
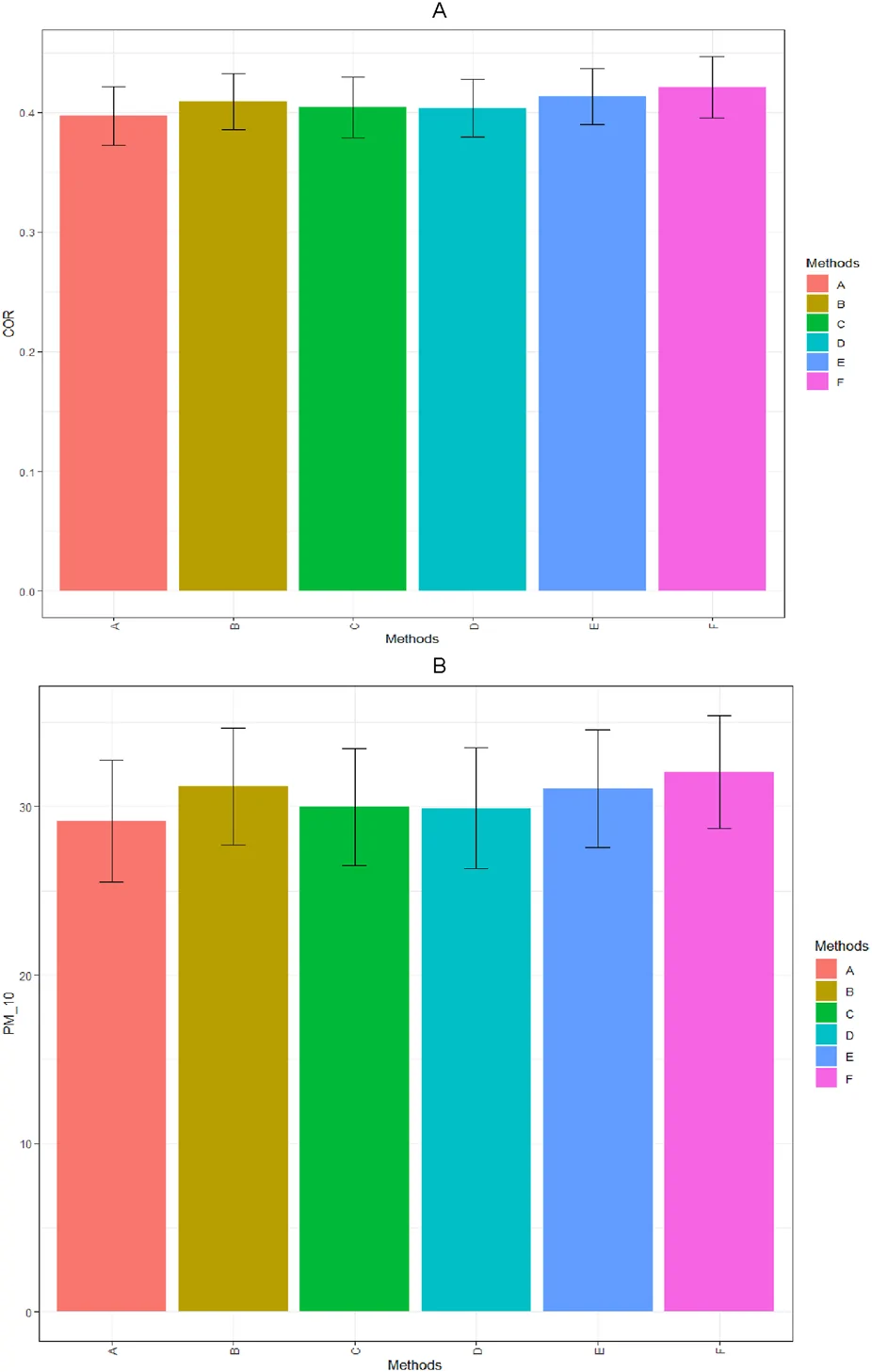
Prediction performance of the six evaluated methods on the Japonica dataset, measured by average Pearson’s correlation [COR; **(A)**] and the proportion of test items whose predicted top 10% lines match the true top 10% [PM_10, **(B)**].

Regarding PM_10, Model F produced the highest predictive value (PM_10 = 32.038), followed closely by Model B (PM_10 = 31.188), which was 2.7% lower than that of Model F (Figure 3B). In contrast, Model A reported the weakest outcome (PM_10 = 29.138; Figure 3B), indicating a 10.0% decline in relation to Model F. Model D showed the second-lowest performance, with a PM_10 of 29.898, corresponding to a 7.2% reduction compared to Model F. More details of the prediction performance for this dataset are given in Supplementary Appendix A, Supplementary Table A2.

### Wheat_1 dataset

The G × E component explained the largest share of the variance across all models, followed by the genotype component (Table 5). When examining the G × E subcomponents (VGE, VGEC, and VGEP), models C, D, E, and F captured a substantially greater proportion of the total variability compared with model B, which included only the conventional G × E interaction term. Specifically, the increases relative to model B were 82.74% for model C, 220.238% for model D, 379.167% for model E, and 466.667% for model F.

**TABLE 5:** Proportion of total variance in the Wheat_1 dataset explained for each variance component in each of the evaluated models. VGE_A = VGE + VGEC + VGEP, which denotes the total proportion of variance explained by the G × E interaction. VEnv= $\sigma_{E}^{2}$ , VG= $\sigma_{g}^{2}$ , VGE= $\sigma_{\text{gE}}^{2}$ , VGEC= $\sigma_{CC}^{2}$ , VGEP= $\sigma_{PP}^{2}$ and VE= $\sigma^{2}.$

Dataset	Methods	VEnv	VG	VGE	VGEC	VGEP	VE	VGE_A
Wheat_1	A	0.460	0.370	-	-	-	0.169	0.000
Wheat_1	B	0.001	0.592	0.168	-	-	0.239	0.168
Wheat_1	C	0.513	0.121	-	0.307	-	0.060	0.307
Wheat_1	D	0.000	0.310	-	-	0.538	0.151	0.538
Wheat_1	E	0.000	0.128	-	0.480	0.325	0.066	0.805
Wheat_1	F	0.000	0.145	0.053	0.465	0.267	0.070	0.784

Regarding COR, Model F demonstrated the highest predictive performance (COR = 0.530), followed closely by Model E (COR = 0.528), which was 0.4% lower than Model F (Figure 4A). In contrast, Model A showed the weakest performance (COR = 0.447; Figure 4A), representing an 18.6% decline relative to Model F. Model B ranked second lowest, with a COR of 0.457, reflecting a 16.0% reduction compared to Model F.

**FIGURE 4:**
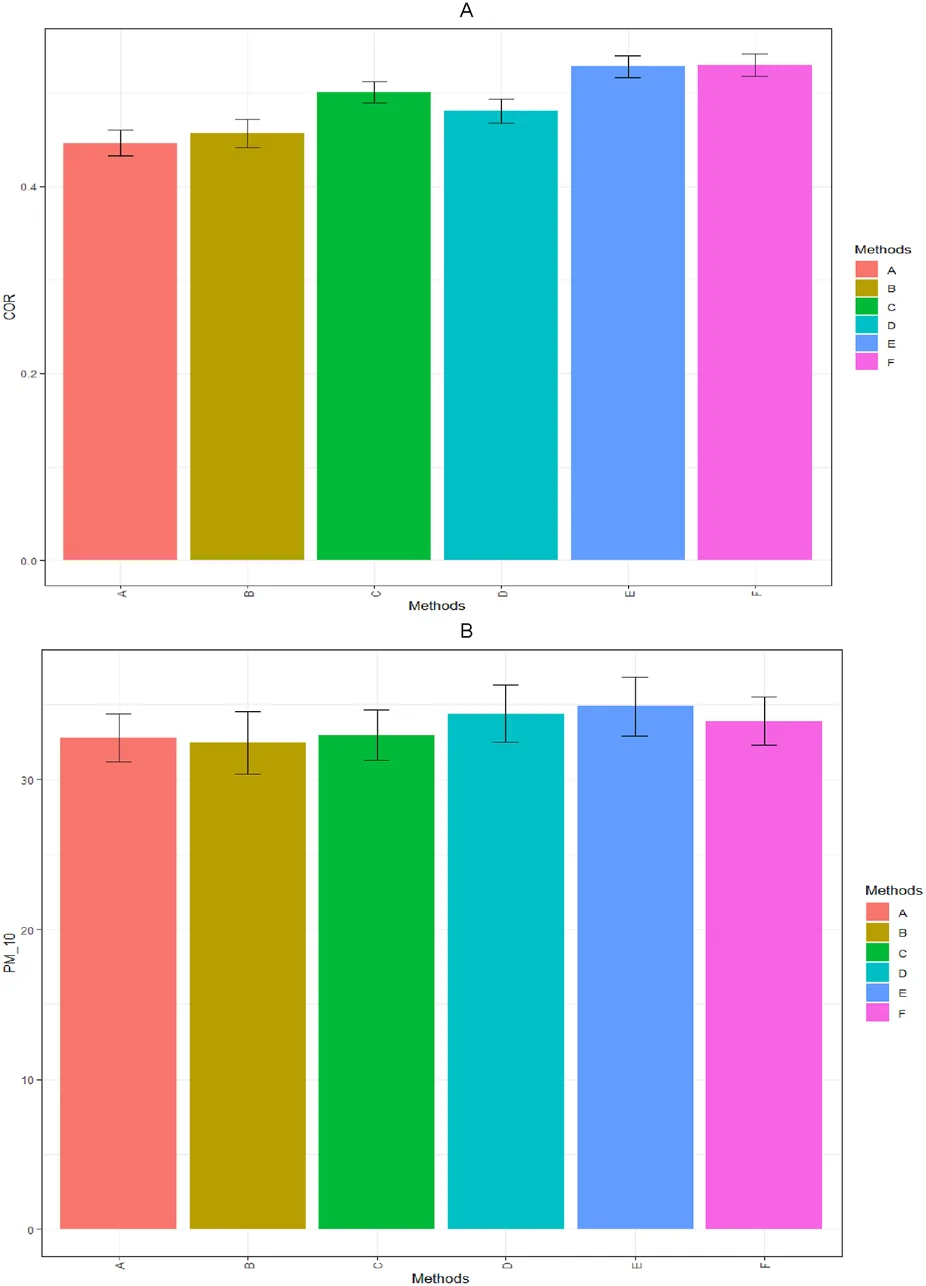
Prediction performance of the six evaluated methods on the Wheat_1 dataset, measured by average Pearson’s correlation [COR; **(A)**] and the proportion of test items whose predicted top 10% lines match the true top 10% [PM_10, **(B)**].

In terms of PM_10, Model E attained the best predictive outcome (PM_10 = 34.879), followed by Model D (PM_10 = 34.411), which was 1.4% below the value for Model E (Figure 4B). On the other hand, Model B had the poorest result (PM_10 = 32.452; Figure 4B), indicating a 7.5% decrease compared to Model E, while Model A exhibited the second-lowest value, with a PM_10 of 32.787–6.4% lower than Model E. More details of the prediction performance for this dataset are given in Supplementary Appendix A, Supplementary Table A2.

### Across datasets

Across the eleven datasets analyzed, the environment component consistently explained the largest proportion of variance, followed by the genotype component (Table 6). For the G × E subcomponents (VGE, VGEC, and VGEP), models C, D, E, and F accounted for a markedly higher share of the total variability compared with model B, which included only the conventional G × E interaction term. In particular, the gains relative to model B were 3.669% for model C, 41.284% for model D, 87.156% for model E, and 122.018% for model F. However, the gains in the proportion of total variance explained in models C and D did not increase execution time, as these models required 16.192% (model C) and 19.021% (model D) less time than model B. In contrast, models E and F showed longer execution times, exceeding that of model B by 24.858% and 92.412%, respectively. The proportion of total variance in the EYT_2, EYT_3, Wheat_2, Wheat_3, Wheat_4, Wheat_5, and Wheat_6 datasets is provided in Supplementary Appendix A, Supplementary Table A1.

**TABLE 6:** Proportion of total variance, across traits and datasets, explained for each variance component in each of the evaluated models. VGE_A = VGE + VGEC + VGEP, which denotes the total proportion of variance explained by the G × E interaction. VEnv= $\sigma_{E}^{2}$ , VG= $\sigma_{g}^{2}$ , VGE= $\sigma_{\text{gE}}^{2}$ , VGEC= $\sigma_{CC}^{2}$ , VGEP= $\sigma_{PP}^{2}$ and VE= $\sigma^{2}$ . Time reports the execution time of each model across traits and datasets.

Dataset	Methods	VEnv	VG	VGE	VGEC	VGEP	Ve	VGE_A	Time
Across_data	A	0.504	0.285	-	-	-	0.211	0.000	96.520
Across_data	B	0.402	0.318	0.109	-	-	0.171	0.109	331.640
Across_data	C	0.486	0.244	-	0.113	-	0.157	0.113	277.942
Across_data	D	0.432	0.255	-	-	0.154	0.160	0.154	268.559
Across_data	E	0.433	0.238	-	0.099	0.106	0.124	0.204	414.080
Across_data	F	0.401	0.257	0.041	0.099	0.101	0.100	0.242	638.115

In relation to COR, model F exhibited the strongest predictive capability (COR = 0.438), followed closely by model E (COR = 0.431), which was 1.6% lower than that of model F (Figure 5A). Conversely, model A recorded the lowest performance (COR = 0.355; Figure 5A), reflecting a 23.4% decrease relative to model F. Model B was the second least effective, with a COR of 0.387, corresponding to a 13.2% reduction compared to model F.

**FIGURE 5:**
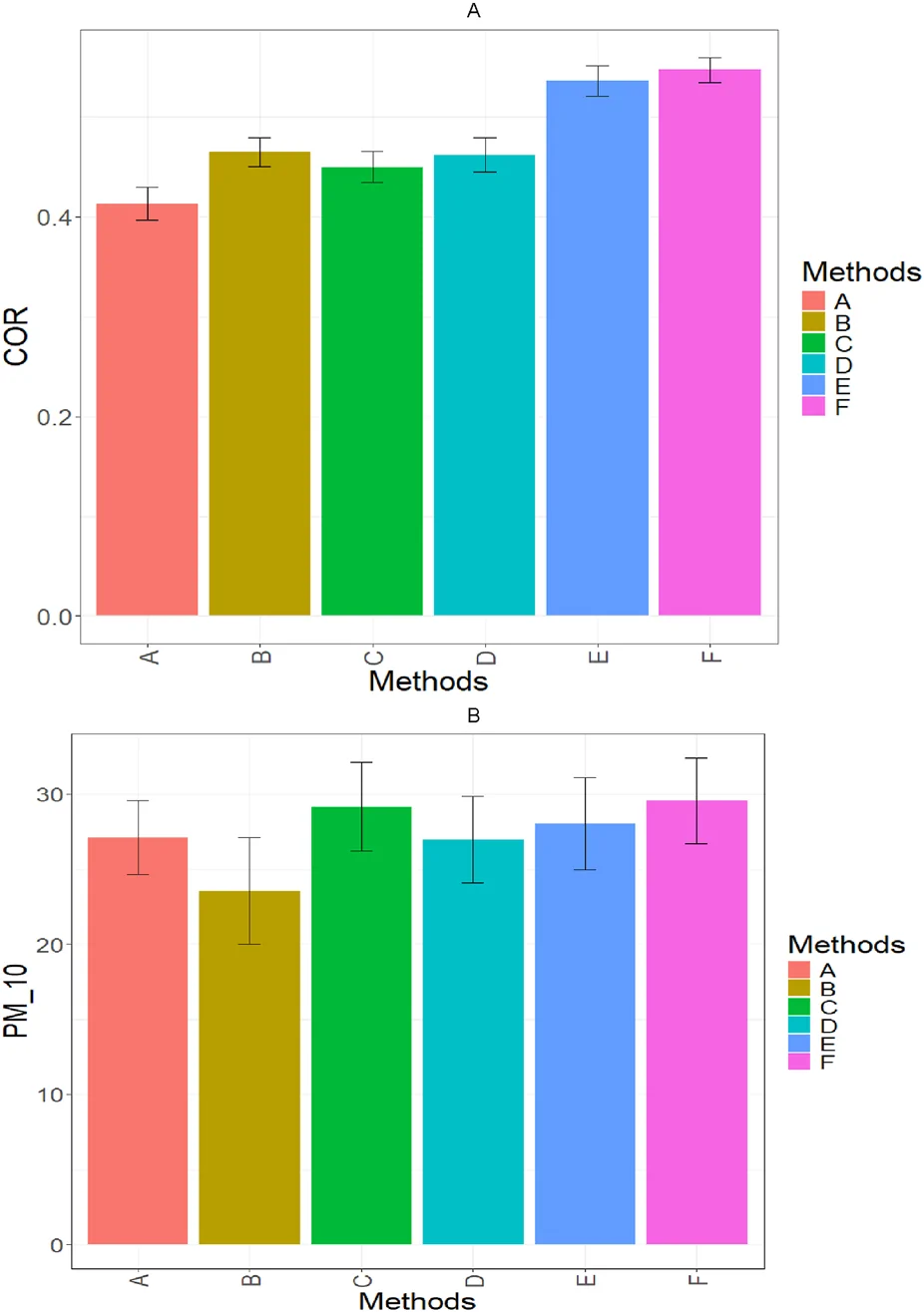
Prediction performance of the six evaluated methods across datasets, measured by average Pearson’s correlation [COR; **(A)**] and the proportion of test items whose predicted top 10% lines match the true top 10% [PM_10, **(B)**].

With respect to PM_10, model F achieved the highest predictive value (PM_10 = 30.117), followed by model E (PM_10 = 29.331), which was 2.7% lower than that of model F (Figure 5B). In contrast, model A yielded the lowest performance (PM_10 = 25.574; Figure 5B), representing a 17.8% decline in comparison to model F. Model D showed the second lowest performance, with a PM_10 of 27.157, which was 10.94% lower than that of model E. More details of the prediction performance across datasets are given in Supplementary Table A2, Supplementary Appendix A, while the prediction performance of the remaining datasets (EYT_2, EYT_3, Wheat_2, Wheat_3, Wheat_4, Wheat_5 and Wheat_6) is provided in Supplementary Appendix B, Supplementary Table B1.

## Discussion

Modeling G × E interaction remains one of the most complex and critical challenges in the development of robust genomic prediction (GP) models. In plant breeding, G × E refers to the differential performance of genotypes across varying environmental conditions, and inadequate modeling of this interaction can lead to biased or inaccurate predictions, reducing the effectiveness of genomic selection in diverse agroecological systems (Crossa et al., 2017).

One of the central difficulties in modeling G × E lies in capturing both environmental variability and the dynamic nature of genotype responses. Traditional GP models often treat G × E as random noise or as a simple additive component, which can oversimplify the interaction and fail to exploit its structured variability, particularly in multi-environment trials (Jarquín et al., 2014). To address this, models incorporating covariance structures derived from genomic and environmental kernels have been proposed (Burgueño et al., 2012), among which the Hadamard (element-wise) product has become a widely used approach.

The Hadamard product captures G × E interaction through joint similarity between genotypes and environments and has a clear covariance interpretation. The matrix-product-derived terms evaluated here can add predictive information under some incidence structures, but they should not be interpreted as uniquely defined biological directions. In particular, the numerical upper/lower split is order-dependent, and the strength and rank of the product can change substantially between balanced and unbalanced genotype-by-environment designs.

We evaluated an alternative framework based on matrix multiplication between genomic and environmental relationship matrices, adapted from Cuevas et al. (2025). The two symmetric terms used in Models C and D are obtained by symmetrizing complementary triangular subsets of the product. They can be included alone or together with the Hadamard interaction term, but their interpretation is numerical and conditional on the chosen record ordering. This limitation is now made explicit rather than attributing intrinsic biological direction to upper versus lower position.

Across the eleven datasets, Model E and especially the combined Model F showed higher predictive performance on average than the Hadamard-only Model B under the implemented random-partition validation. The average COR difference between Models F and B was 13.2%, and the corresponding average PM_10 difference was 10.3%. These are descriptive average improvements under the analyzed partitions, not evidence that Model F is universally or statistically significantly superior in every dataset. Formal paired resampling on identical folds would be required for inferential claims about model differences.

The average performance of Model F suggests that the conventional Hadamard term and the additional matrix-product-derived terms may carry complementary predictive information. However, the present results do not establish that the triangular components correspond to distinct biological mechanisms or uniquely identifiable portions of G × E variance. This distinction is particularly important because kernels derived from the same underlying matrices may be correlated, and because balanced designs can produce low-rank or degenerate matrix products.

The contrast between balanced and unbalanced datasets is especially informative. In a balanced experiment, the matrix product may be dominated by one or a few directions, whereas imbalance, replication patterns, and missing genotype-by-environment cells can increase its effective rank. A low-rank term may nevertheless improve prediction by contributing a stable covariance direction that is not fully represented by the other fitted kernels. Such a gain should be interpreted as evidence of predictive complementarity, not as proof that the added term captures a distinct high-dimensional G × E mechanism. This distinction is important when transferring the method to other breeding programs.

The novelty of this work lies in evaluating the matrix-product framework of Cuevas et al. (2025) in the context of G × E prediction across a broad collection of wheat and rice datasets. The revision narrows the interpretation of the triangular kernels and separates their empirical predictive contribution from questions of representation invariance and covariance construction. A general decomposition whose individual components retain their identity under arbitrary relabeling of the same biological records remains an important direction for subsequent methodological research.

Accurately modeling G × E interactions is essential for improving the predictive performance of genomic selection, as environmental factors play a major role in determining phenotypic expression and genotype adaptability. This challenge becomes even more critical under climate change, where increasing environmental variability and uncertainty reduce the effectiveness of traditional models (Costa-Neto et al., 2021; Elias et al., 2016). In such contexts, more flexible modeling strategies are required to capture heterogeneous and complex interaction patterns.

The matrix-product formulation provides an additional way to construct predictors from genomic and environmental relationship information. Its usefulness is empirical and depends on the incidence structure, kernel construction, covariance regularization, and validation problem. These dependencies should be reported explicitly when the method is transferred to other breeding datasets.

In parallel, recent advances in machine learning, deep learning, graph-based models, and multi-omics integration offer alternative high-dimensional approaches for genomic prediction. These approaches can capture nonlinear structure but raise their own questions about interpretability, data requirements, and computational cost (Montesinos-López et al., 2021; Wang et al., 2026). The matrix-product approach considered here remains within a mixed-model framework, which facilitates covariance-based interpretation while making its structural assumptions explicit.

In summary, G × E interaction is a fundamental and complex component of genomic prediction that requires flexible and structured modeling approaches. The results of this study demonstrate that combining conventional and matrix-product-based covariance structures provides a more complete and effective representation of G × E interaction. This perspective opens new opportunities for developing more flexible and biologically meaningful covariance models for multi-environment genomic prediction in plant breeding. Importantly, the proposed framework should not be interpreted as a hybrid kernel approach, but rather as an extension of matrix-product-based covariance constructions to the specific problem of G × E.

## Conclusion

In this study, we evaluated matrix-product-derived covariance terms as additions or alternatives to the conventional Hadamard G × E interaction kernel. Across 11 datasets, the combined formulation showed higher predictive performance on average under the implemented validation scheme, but the magnitude of improvement varied among datasets and metrics. The revision also clarifies important structural limitations: the upper/lower triangular partition is tied to record ordering, balanced centered designs can yield low-rank or degenerate products, and symmetric matrix-product-derived kernels require explicit PSD validation before use as covariance structures. Thus, the present contribution is an empirical assessment of the predictive information available from these matrix-product-derived terms, not a claim that their positional decomposition is an intrinsic biological direction or universally optimal covariance representation. Permutation-equivariant decompositions and stricter prospective validation provide important directions for further methodological development.

## Data Availability

The datasets and code presented in this study can be found in the following online repository: https://github.com/osval78/New_GE_Framework.
