# Supplementary material for: A novel matrix multiplication framework for modeling genotype-by-environment interaction in genomic prediction
Source: Front Genet. 2026 Sep 29;17:1961511. doi: 10.3389/fgene.2026.1961511 (PMC13659291; doi:10.3389/fgene.2026.1961511)
Supplement: Supplementary file 1 [file Supplementaryfile1.docx]

**Supplementary Material**

**Supplementary Note 1. Positive Semidefiniteness and Construction of Covariance Structures in Models A–F**

This Supplement provides the mathematical justification and diagnostic requirements for the covariance structures used in Models A–F. Because the matrix-product-derived kernels are symmetric by construction but are not guaranteed to be positive semidefinite, their spectra must be examined explicitly. The revision also documents two issues raised during peer review: dependence of numerical triangular extraction on observation ordering, and degeneracy of the matrix product under some balanced, centered designs.

**A. Genomic relationship matrix** $\mathbf{G}$**(Model A)**

The genomic relationship matrix is defined as:

**G** = 1/p **ZZ^T^**

where $\mathbf{Z}$ is the standardized marker matrix. Because $\mathbf{G}$ is a Gram matrix, for any vector $\mathbf{x}$:

**x^T^Gx** = 1/p ‖**Z^T^x**‖^2^ ≥ 0

Thus, $\mathbf{G}$ is symmetric and positive semidefinite.

**B. Environmental kernel** $\mathbf{K}_{\mathbf{E}}$

The environmental kernel is constructed as:

**K_E_** = **WW^T^** / I

where $\mathbf{W}$ contains environmental covariates or design matrix of environments in the absence of environmental covariates. Thus, $\mathbf{K}_{\mathbf{E}}$ is also a Gram matrix and is symmetric and PSD. Here, I denotes the number of environments (or environmental covariates).

**C. Genetic kernel at the observation level:**

**K_g_** = **Z_g_GZ_g_^T^**

To show that ${\mathbf{K}_{\mathbf{g}}\mathbf{=Z}}_{\mathbf{g}}\mathbf{G}\mathbf{Z}_{\mathbf{g}}^{T}$ is PSD, we need to show that for any vector $\mathbf{x}$, $\mathbf{x}^{T}\mathbf{K}_{\mathbf{g}}\mathbf{x}\geq0.$

Let $\mathbf{x}$ be any real vector of appropriate dimension. Then:

**x^T^K_g_x** = **x^T^Z_g_GZ_g_^T^x** ≥ 0

$$\mathbf{x}^{T}\mathbf{K}_{\mathbf{g}}\mathbf{x}=\mathbf{x}^{T}\mathbf{Z}_{\mathbf{g}}\mathbf{G}\mathbf{Z}_{\mathbf{g}}^{T}\mathbf{x.}$$

Define $\mathbf{y}=\mathbf{Z}_{\mathbf{g}}^{T}\mathbf{x}$, then the quadratic form becomes $\mathbf{x}^{T}\mathbf{K}_{\mathbf{g}}\mathbf{x}=\mathbf{y}^{T}\mathbf{G}\mathbf{y}$.

Since $\mathbf{G}$ is a genomic relationship matrix and is PSD by construction, for any vector $\mathbf{y}$, $\mathbf{y}^{T}\mathbf{G}\boldsymbol{y\geq0}$**.** Thus

$$\mathbf{x}^{T}\mathbf{K}_{\mathbf{g}}\mathbf{x}=\mathbf{y}^{T}\mathbf{G}\boldsymbol{y\geq0}$$

This holds for all vectors $\mathbf{x}$, so $\mathbf{K}_{\mathbf{g}}\mathbf{=}\mathbf{Z}_{\mathbf{g}}\mathbf{G}\mathbf{Z}_{\mathbf{g}}^{T}$ is PSD.

**D. Hadamard Kernel** $\mathbf{K}_{\mathbf{g}}^{\circ}\mathbf{K}_{\mathbf{E}}$ **(Model B)**

The **Schur–Hadamard theorem** states:

***A*** and ***B*** are PSD matrices of the same dimension, then ***A*** ∘ ***B*** is PSD. Where ∘ denotes the Hadamard product. Since both $\mathbf{K}_{\mathbf{g}}$ and $\mathbf{K}_{\mathbf{E}}$ are PSD, their Hadamard product $\mathbf{K}_{\mathbf{g}}^{\circ}\mathbf{K}_{\mathbf{E}}$ is also PSD. This ensures that the covariance matrix for the G$\times$E interaction in Model B is valid.

**E. Matrix-product-based kernels (Models C and D)**

Let:

$\mathbf{M}=\mathbf{K}_{\mathbf{E}}\mathbf{K}_{\mathbf{g}}$

Although both $\mathbf{K}_{\mathbf{E}}$and $\mathbf{K}_{\mathbf{g}}$ are PSD, their matrix multiplication product $\mathbf{M}$is not necessarily symmetric or PSD. Define the upper‑triangular operator as:

**M** = **K_E_K_g_**

$$\mathbf{U}T_{ij}=\left\{ \begin{aligned} \mathbf{M}_{ij}, &i\leq j, \\ 0, &i>j. \end{aligned} \right.$$

The diagonal operator as:

$$\mathbf{D}_{ij}=\left\{ \begin{aligned} \mathbf{M}_{ii}, &i=j, \\ 0, &i\neq j. \end{aligned} \right.$$

And the lower-triangular operator as:

$$\mathbf{U}_{ij}=\left\{ \begin{aligned} \mathbf{M}_{ij}, &i<j, \\ 0, &i\geq j. \end{aligned} \right.$$

Thus, the full matrix, $\mathbf{M}$**,** satisfies:

$$\mathbf{M}=\mathbf{L+D+U}\boldsymbol{.}$$

***Model C (upper-triangular-based kernel)***

The covariance matrix used in Model C is

**K_CC_** = **D** + **U** + **U^T^**

where **D** is the diagonal part, and **U** is the strictly upper-triangular part. This construction preserves the diagonal and upper-triangular elements of **M** and reflects the upper-triangular information across the diagonal, thereby ensuring that **K_CC_** is symmetric (Cuevas et al., 2025). However, symmetry alone does not guarantee positive semidefiniteness. Therefore, **K_CC_** is treated as an empirically constructed kernel whose spectrum is examined explicitly; when negative eigenvalues are detected, the PSD projection described below is applied before the kernel is used as a variance-covariance structure.

**Model D (lower-triangular-based kernel)**

Similarly, the covariance matrix in Model D is based on the **lower‑triangular** component:

**K_PP_** = **D** + **L** + **L^T^**

where **L** is the strictly lower-triangular part. The construction of **K_PP_** preserves the diagonal **D** and lower-triangular **L** elements of ***M***, while reflecting the lower-triangular information across the diagonal to ensure symmetry (Cuevas et al., 2025). Accordingly, ***K_PP_*** is treated as an empirical kernel whose positive semidefiniteness is verified through eigendecomposition of its spectrum as described above.

**Important remark**

The upper- and lower-triangular symmetrizations are symmetric, but positive semidefiniteness is not guaranteed analytically. For each constructed kernel **K**, use the spectral decomposition **K = VΛVᵀ** and define **Λ+ = diag{max(λi,0)}** after applying a numerical tolerance **ᵀ**. Eigenvalues **λi < -ᵀ** are treated as negative and set to zero; eigenvalues within ±τ are treated as numerical zero. The corrected kernel is **K+ = VΛ+Vᵀ**. This PSD projection does not, in general, preserve the original diagonal, and the diagonal preservation is therefore not claimed.

**K** = **VΛV^T^**, **Λ_+_** = diag{max(λ_i_,0)}, **K_+_** = **VΛ_+_V^T^**

For each dataset and each matrix-product-derived kernel, the reproducibility report should include at minimum: the minimum eigenvalue before correction; the number and proportion of negative eigenvalues and the negative spectral mass **Σ|λi|** over **λi<0** relative to **Σ|λi|**; and a sensitivity comparison of predictions before versus after correction whenever the uncorrected matrix can be fit safely for diagnostic purposes. These quantities make the magnitude of the spectral modification transparent rather than treating PSD correction as a purely technical step.

**F. Combined kernels (Models E and F)**

**Model E**

Finally, the two components of the $\boldsymbol{G\times E}$ interaction are **K_CC_** and **K_PP_**

**Model F**

For Model F, the three components for modeling the $\mathbf{G}\times E$ interaction are **K_E_** ∘ **K_g_**, **KCC**, and **K_PP_**. After the spectral correction described above, each kernel component used in Models E and F is positive semidefinite; therefore, the resulting covariance matrices are valid for modeling.

**G. Conceptual implication**

In contrast to the Hadamard kernel, the matrix-product-based kernels retain different subsets of the information contained in the noncommutative product $\mathbf{K}_{\mathbf{E}}\mathbf{K}_{\mathbf{g}}$. The upper and lower triangular constructions may therefore represent complementary covariance patterns that are not explicitly captured by the element-wise product. Their potential predictive advantage is evaluated empirically in Models E and F.

**Conclusion**

The covariance matrices used in Models A and B are symmetric and positive semidefinite by construction. The matrix-product-based kernels introduced in Models C and D are symmetric but are not guaranteed to be positive semidefinite. Therefore, their spectra are examined, and a positive-semidefinite projection is applied whenever negative eigenvalues are detected. The corrected kernels are subsequently used in Models C–F, ensuring that all covariance structures employed in the multivariate normal models are mathematically valid. These matrix-product-based kernels provide alternative and potentially complementary representations of G×E covariance, whose predictive value is assessed empirically.

**Supplementary Note 2. Observation ordering, balanced incidence, and matrix-product rank**

Ordering. Let $\mathbf{M}=\mathbf{K}_{\mathbf{E}}\mathbf{K}_{\mathbf{g}}$ denote the observation-level matrix product. Under a simultaneous permutation Q of the same records, the full product transforms as **M* = QMQᵀ.** However, the numerical upper-triangular operator **U(·)** generally does not satisfy **U(QMQᵀ) = QU(M)Qᵀ**; the analogous statement holds for the lower-triangular operator. Thus, sorting records before construction produces a deterministic convention but does not make the separate triangular components permutation-equivariant. The current manuscript therefore interprets these kernels as positional numerical constructions and does not assign intrinsic biological direction to upper versus lower position.

**M^*^** = **QMQ^T^**, 𝒰(**QMQ^T^**) ≠ **Q**𝒰(**M**)**Q^T^**

Balanced incidence. Let **Z_E_** and **Z_g_** be observation-to-environment and observation-to-genotype incidence matrices. The observation-level kernels can be written schematically as **K_E_**= **Z_E_Z_E_^T^** and **K_g_**= **Z_g_GZ_g_^T^**, so that **K_E_K_g_** contains the cross-incidence factor **Z_E_^T^Z_g_**. In a complete balanced genotype-by-environment design with one observation for every genotype in every environment, **Z_E_^T^Z_g_** is an all-ones matrix. Consequently, the product can collapse to a low-rank form. If the genomic relationship matrix is derived from centered markers, **G** is zero or numerically close to zero under common centering conventions; multiplication by an all-ones cross-incidence factor can therefore make **K_E_K_g_** zero or nearly zero. Departures from complete balance, unequal replication, missing genotype-environment cells, or alternative centering can prevent this cancellation and increase the rank and magnitude of the product.

**Z_E_^T^Z_g_** = **J**, **K_g_1** = **0** ⇒ **K_E_K_g_** may be zero or low-rank

This result explains why experimental balance is not a peripheral detail of the method. The rank and spectral content of M, and therefore of the triangular kernels derived from $\mathbf{M}$, should be reported for each dataset. A predictive gain obtained from a very low-rank product may still be empirically useful, but it should not automatically be interpreted as a rich G×E covariance pattern.

Low-rank products and predictive utility. The rank of a product satisfies rank($\mathbf{K}_{\mathbf{E}}\mathbf{K}_{\mathbf{g}}$) ≤ min{rank($\mathbf{K}_{\mathbf{E}}$), rank($\mathbf{K}_{\mathbf{g}}$)}. In complete balanced designs, additional incidence constraints can reduce the effective rank much further, potentially to one dominant direction. Such a kernel can still contribute to prediction if that direction is aligned with phenotype-associated covariance or supplies regularization complementary to other model terms. Nevertheless, prediction gain alone is not sufficient to label that low-rank direction as a distinct G×E mechanism. Accordingly, rank, eigenvalue concentration, and kernel overlap should be examined together with prediction metrics.

Dataset-level diagnostic reporting. For reproducibility and interpretation, the analysis output should list the dimensions and numerical rank of the full matrix product, the rank of each symmetrized triangular kernel, the leading eigenvalues or cumulative spectral concentration, and the overlap/correlation among fitted kernels. These quantities are particularly important for comparing balanced datasets such as Indica or EYT-type trials with unbalanced datasets, because the same predictive gain can arise from covariance structures with very different effective dimensionality.

rank($\mathbf{K}_{\mathbf{E}}\mathbf{K}_{\mathbf{g}}$) ≤ min{rank($\mathbf{K}_{\mathbf{E}}$), rank($\mathbf{K}_{\mathbf{g}}$)}

**Supplementary Note 3. Kernel overlap, identifiability, and MCMC diagnostics**

Models E and F contain two or three G×E kernels constructed from overlapping sources of genotype and environment information. These kernels need not span orthogonal covariance spaces. Therefore, posterior variance components can trade off against one another, especially when kernels are highly correlated or rank deficient. The fitted quantities VGEC and VGEP should be interpreted as model-specific variance parameters, not as uniquely attributable or orthogonal biological fractions of G×E variation.

For each dataset, recommended diagnostics are: pairwise Frobenius kernel correlations after common scaling; numerical rank; leading eigenvalue proportions; condition diagnostics for the combined kernel basis; and the exact scaling applied before fitting. For Bayesian fits, convergence should be documented with trace plots or equivalent summaries, effective sample sizes, Monte Carlo error (or comparable diagnostics), and, where feasible, multiple-chain agreement. These diagnostics are particularly important for Models E and F because several random effects are derived from the same underlying matrices.

**Supplementary Note 4. Validation and reproducibility requirements**

We implemented a strict ULTE design since the entire genotype identities across all tested environments were some part assigned for the training and the remaining for the testing, that is, one genotype that appeared in the training not appeared in the testing set.

The resubmission include the complete R code, including fold generation, random seeds, BGLR iterations, burn-in, thinning, PSD projection, kernel scaling, package versions, hardware information, data paths, and scripts that regenerate all tables and figures. This requirement is separate from the mathematical validity of the model but essential for independent reproduction of the empirical results.

**Supplementary References**

Higham, N. J. (1988). Computing a nearest symmetric positive semidefinite matrix. *Linear Algebra and its Applications*, *103*, 103–118. doi: 10.1016/0024-3795(88)90223-6
