# Supplementary material for: A novel matrix multiplication framework for modeling genotype-by-environment interaction in genomic prediction
Source: Front Genet. 2026 Sep 29;17:1961511. doi: 10.3389/fgene.2026.1961511 (PMC13659291; doi:10.3389/fgene.2026.1961511)
Supplement: Supplementary file 2 [file Supplementaryfile2.docx]

**Appendix A**

**Table A1**. Proportion of total variance in EYT_2, EYT_3, Wheat_2, Wheat_3, Wheat_4, Wheat_5, and Wheat_6 datasets, explained for each variance component in each of the evaluated models. VGE_A= VGE+ VGEC+ VGEP, which denotes the total proportion of variance explained by the G×E interaction. VEnv=$\sigma_{\mathbf{E}}^{2}$, VG=$\sigma_{\mathbf{g}}^{2}$, VGE=$\sigma_{\mathbf{gE}}^{2}$, VGEC=$\sigma_{CC}^{2}$, VGEP=$\sigma_{PP}^{2}$ and VE=$\sigma^{2}.$

| Dataset | Methods | VEnv | VG | VGE | VGEC | VGEP | Ve | VGE_A |
| --- | --- | --- | --- | --- | --- | --- | --- | --- |
| EYT_2 | A | 0.724 | 0.197 | NA | NA | NA | 0.079 | 0.000 |
| EYT_2 | B | 0.677 | 0.230 | 0.032 | NA | NA | 0.060 | 0.032 |
| EYT_2 | C | 0.745 | 0.177 | NA | 0.008 | NA | 0.069 | 0.008 |
| EYT_2 | D | 0.715 | 0.201 | NA | NA | 0.008 | 0.076 | 0.008 |
| EYT_2 | E | 0.709 | 0.207 | NA | 0.012 | 0.011 | 0.061 | 0.023 |
| EYT_2 | F | 0.678 | 0.233 | 0.021 | 0.010 | 0.009 | 0.049 | 0.040 |
| EYT_3 | A | 0.801 | 0.139 | NA | NA | NA | 0.060 | 0.000 |
| EYT_3 | B | 0.771 | 0.167 | 0.025 | NA | NA | 0.038 | 0.025 |
| EYT_3 | C | 0.808 | 0.141 | NA | 0.006 | NA | 0.045 | 0.006 |
| EYT_3 | D | 0.807 | 0.136 | NA | NA | 0.006 | 0.052 | 0.006 |
| EYT_3 | E | 0.794 | 0.151 | NA | 0.009 | 0.009 | 0.038 | 0.017 |
| EYT_3 | F | 0.780 | 0.161 | 0.015 | 0.007 | 0.007 | 0.031 | 0.029 |
| Wheat_2 | A | 0.394 | 0.304 | NA | NA | NA | 0.301 | 0.000 |
| Wheat_2 | B | 0.001 | 0.385 | 0.245 | NA | NA | 0.370 | 0.245 |
| Wheat_2 | C | 0.190 | 0.190 | NA | 0.454 | NA | 0.167 | 0.454 |
| Wheat_2 | D | 0.000 | 0.207 | NA | NA | 0.580 | 0.213 | 0.580 |
| Wheat_2 | E | 0.000 | 0.115 | NA | 0.432 | 0.351 | 0.101 | 0.784 |
| Wheat_2 | F | 0.000 | 0.135 | 0.045 | 0.451 | 0.261 | 0.108 | 0.757 |
| Wheat_3 | A | 0.448 | 0.222 | NA | NA | NA | 0.330 | 0.000 |
| Wheat_3 | B | 0.001 | 0.226 | 0.495 | NA | NA | 0.278 | 0.495 |
| Wheat_3 | C | 0.273 | 0.113 | NA | 0.482 | NA | 0.132 | 0.482 |
| Wheat_3 | D | 0.000 | 0.085 | NA | NA | 0.799 | 0.115 | 0.799 |
| Wheat_3 | E | 0.000 | 0.053 | NA | 0.321 | 0.578 | 0.048 | 0.899 |
| Wheat_3 | F | 0.000 | 0.057 | 0.048 | 0.312 | 0.536 | 0.047 | 0.896 |
| Wheat_4 | A | 0.019 | 0.499 | NA | NA | NA | 0.482 | 0.000 |
| Wheat_4 | B | 0.000 | 0.436 | 0.192 | NA | NA | 0.372 | 0.192 |
| Wheat_4 | C | 0.000 | 0.170 | NA | 0.647 | NA | 0.183 | 0.647 |
| Wheat_4 | D | 0.000 | 0.182 | NA | NA | 0.663 | 0.155 | 0.663 |
| Wheat_4 | E | 0.000 | 0.090 | NA | 0.379 | 0.466 | 0.065 | 0.844 |
| Wheat_4 | F | 0.000 | 0.091 | 0.008 | 0.361 | 0.474 | 0.066 | 0.843 |
| Wheat_5 | A | 0.010 | 0.552 | NA | NA | NA | 0.439 | 0.000 |
| Wheat_5 | B | 0.000 | 0.498 | 0.197 | NA | NA | 0.305 | 0.197 |
| Wheat_5 | C | 0.001 | 0.297 | NA | 0.493 | NA | 0.209 | 0.493 |
| Wheat_5 | D | 0.000 | 0.207 | NA | NA | 0.640 | 0.153 | 0.640 |
| Wheat_5 | E | 0.000 | 0.107 | NA | 0.366 | 0.459 | 0.069 | 0.824 |
| Wheat_5 | F | 0.000 | 0.112 | 0.018 | 0.362 | 0.438 | 0.070 | 0.818 |
| Wheat_6 | A | 0.742 | 0.138 | NA | NA | NA | 0.120 | 0.000 |
| Wheat_6 | B | 0.002 | 0.160 | 0.462 | NA | NA | 0.377 | 0.462 |
| Wheat_6 | C | 0.539 | 0.084 | NA | 0.292 | NA | 0.085 | 0.292 |
| Wheat_6 | D | 0.242 | 0.121 | NA | NA | 0.522 | 0.115 | 0.522 |
| Wheat_6 | E | 0.394 | 0.031 | NA | 0.264 | 0.270 | 0.041 | 0.534 |
| Wheat_6 | F | 0.000 | 0.030 | 0.037 | 0.398 | 0.468 | 0.068 | 0.903 |

**Table A2**. Prediction performance of the six evaluated methods on EYT_1 Indica, Japonica, Wheat_1, and across datasets, measured by average Pearson’s correlation (COR) and the proportion of matching in the top 10% of lines (PM_10). SE_COR denotes the standard error (SE) for the COR metric, while SE_PM_10 is the SE for the PM_10 metric.

| Dataset | Methods | COR | SE_COR | PM_10 | SE_PM_10 |
| --- | --- | --- | --- | --- | --- |
| EYT_1 | A | 0.317 | 0.012 | 22.452 | 1.61 |
| EYT_1 | B | 0.356 | 0.011 | 25.369 | 1.582 |
| EYT_1 | C | 0.352 | 0.013 | 23.718 | 1.733 |
| EYT_1 | D | 0.342 | 0.011 | 23.542 | 1.709 |
| EYT_1 | E | 0.378 | 0.011 | 24.968 | 1.553 |
| EYT_1 | F | 0.395 | 0.011 | 26.122 | 1.625 |
| Indica | A | 0.359 | 0.016 | 24.069 | 2.494 |
| Indica | B | 0.376 | 0.019 | 25.343 | 2.644 |
| Indica | C | 0.372 | 0.019 | 23.431 | 2.561 |
| Indica | D | 0.38 | 0.017 | 24.657 | 2.497 |
| Indica | E | 0.399 | 0.019 | 25.833 | 2.479 |
| Indica | F | 0.405 | 0.02 | 27.255 | 2.757 |
| Japonica | A | 0.397 | 0.024 | 29.138 | 3.627 |
| Japonica | B | 0.409 | 0.024 | 31.188 | 3.459 |
| Japonica | C | 0.404 | 0.025 | 29.965 | 3.456 |
| Japonica | D | 0.404 | 0.024 | 29.898 | 3.573 |
| Japonica | E | 0.413 | 0.023 | 31.072 | 3.508 |
| Japonica | F | 0.421 | 0.026 | 32.038 | 3.358 |
| Wheat_1 | A | 0.447 | 0.014 | 32.787 | 1.612 |
| Wheat_1 | B | 0.457 | 0.015 | 32.452 | 2.079 |
| Wheat_1 | C | 0.501 | 0.011 | 32.98 | 1.69 |
| Wheat_1 | D | 0.481 | 0.013 | 34.411 | 1.895 |
| Wheat_1 | E | 0.528 | 0.012 | 34.879 | 1.953 |
| Wheat_1 | F | 0.53 | 0.012 | 33.902 | 1.601 |
| Acrossdata | A | 0.355 | 0.014 | 25.574 | 2.062 |
| Acrossdata | B | 0.387 | 0.014 | 27.308 | 2.103 |
| Acrossdata | C | 0.39 | 0.015 | 27.226 | 2.121 |
| Acrossdata | D | 0.391 | 0.014 | 27.157 | 2.109 |
| Acrossdata | E | 0.431 | 0.014 | 29.331 | 2.092 |
| Acrossdata | F | 0.438 | 0.014 | 30.117 | 2.113 |

**Appendix B**

**Table B1**. Prediction performance of the six evaluated methods on EYT_2, EYT_3, Wheat_2, Wheat_3, Wheat_4, Wheat_5, and Wheat_6 datasets, measured by average Pearson’s correlation (COR) and the proportion of matching in the top 10% lines (PM_10). SE_COR denotes the standard error (SE) for the COR metric, while SE_PM_10 is the SE for the PM_10 metric.

| Dataset | Methods | COR | SE_COR | PM_10 | SE_PM_10 |
| --- | --- | --- | --- | --- | --- |
| EYT_2 | A | 0.371 | 0.012 | 25.179 | 1.678 |
| EYT_2 | B | 0.397 | 0.011 | 26.205 | 1.539 |
| EYT_2 | C | 0.405 | 0.011 | 26.628 | 1.655 |
| EYT_2 | D | 0.397 | 0.012 | 25.808 | 1.753 |
| EYT_2 | E | 0.436 | 0.011 | 28.449 | 1.809 |
| EYT_2 | F | 0.44 | 0.011 | 28.897 | 1.605 |
| EYT_3 | A | 0.311 | 0.012 | 23.776 | 1.405 |
| EYT_3 | B | 0.378 | 0.01 | 27.337 | 1.496 |
| EYT_3 | C | 0.377 | 0.01 | 27.827 | 1.407 |
| EYT_3 | D | 0.371 | 0.011 | 27.357 | 1.525 |
| EYT_3 | E | 0.442 | 0.01 | 31.52 | 1.545 |
| EYT_3 | F | 0.452 | 0.01 | 32.092 | 1.695 |
| Wheat_2 | A | 0.287 | 0.011 | 27.343 | 1.968 |
| Wheat_2 | B | 0.305 | 0.01 | 30.283 | 1.89 |
| Wheat_2 | C | 0.301 | 0.011 | 27.721 | 1.809 |
| Wheat_2 | D | 0.332 | 0.011 | 29.145 | 1.695 |
| Wheat_2 | E | 0.351 | 0.01 | 29.923 | 1.451 |
| Wheat_2 | F | 0.34 | 0.011 | 31.492 | 1.758 |
| Wheat_3 | A | 0.403 | 0.009 | 24.03 | 1.503 |
| Wheat_3 | B | 0.441 | 0.009 | 25.593 | 1.35 |
| Wheat_3 | C | 0.452 | 0.01 | 27.501 | 1.842 |
| Wheat_3 | D | 0.481 | 0.009 | 28.384 | 1.559 |
| Wheat_3 | E | 0.543 | 0.008 | 32.43 | 1.378 |
| Wheat_3 | F | 0.543 | 0.008 | 32.139 | 1.751 |
| Wheat_4 | A | 0.268 | 0.014 | 26.597 | 1.33 |
| Wheat_4 | B | 0.315 | 0.011 | 25.194 | 1.577 |
| Wheat_4 | C | 0.405 | 0.012 | 34.505 | 1.975 |
| Wheat_4 | D | 0.397 | 0.011 | 31.791 | 1.194 |
| Wheat_4 | E | 0.51 | 0.007 | 39.138 | 1.443 |
| Wheat_4 | F | 0.511 | 0.007 | 38.468 | 1.505 |
| Wheat_5 | A | 0.388 | 0.009 | 28.581 | 1.456 |
| Wheat_5 | B | 0.41 | 0.009 | 31.147 | 1.347 |
| Wheat_5 | C | 0.398 | 0.009 | 29.736 | 1.629 |
| Wheat_5 | D | 0.446 | 0.008 | 30.348 | 1.395 |
| Wheat_5 | E | 0.456 | 0.009 | 30.813 | 1.547 |
| Wheat_5 | F | 0.453 | 0.009 | 31.869 | 1.313 |
| Wheat_6 | A | 0.413 | 0.017 | 27.124 | 2.48 |
| Wheat_6 | B | 0.465 | 0.015 | 23.568 | 3.545 |
| Wheat_6 | C | 0.45 | 0.016 | 29.167 | 2.945 |
| Wheat_6 | D | 0.462 | 0.017 | 26.968 | 2.871 |
| Wheat_6 | E | 0.536 | 0.015 | 28.062 | 3.06 |
| Wheat_6 | F | 0.547 | 0.013 | 29.568 | 2.853 |
